# Supplementary material for: Splice-Junction-Based Mapping of Alternative Isoforms in the Human Proteome
Source: Cell Rep. Author manuscript; Available in PMC 2020 Jan 15. (PMC6961840; doi:10.1016/j.celrep.2019.11.026)
Supplement: 3 [file NIHMS1546469-supplement-3.zip › DF2/PXD009737/TestisGluC-27-O00499-IRVNHEPEPAGGATPGATLPKSPSQPAEASE.pdf]

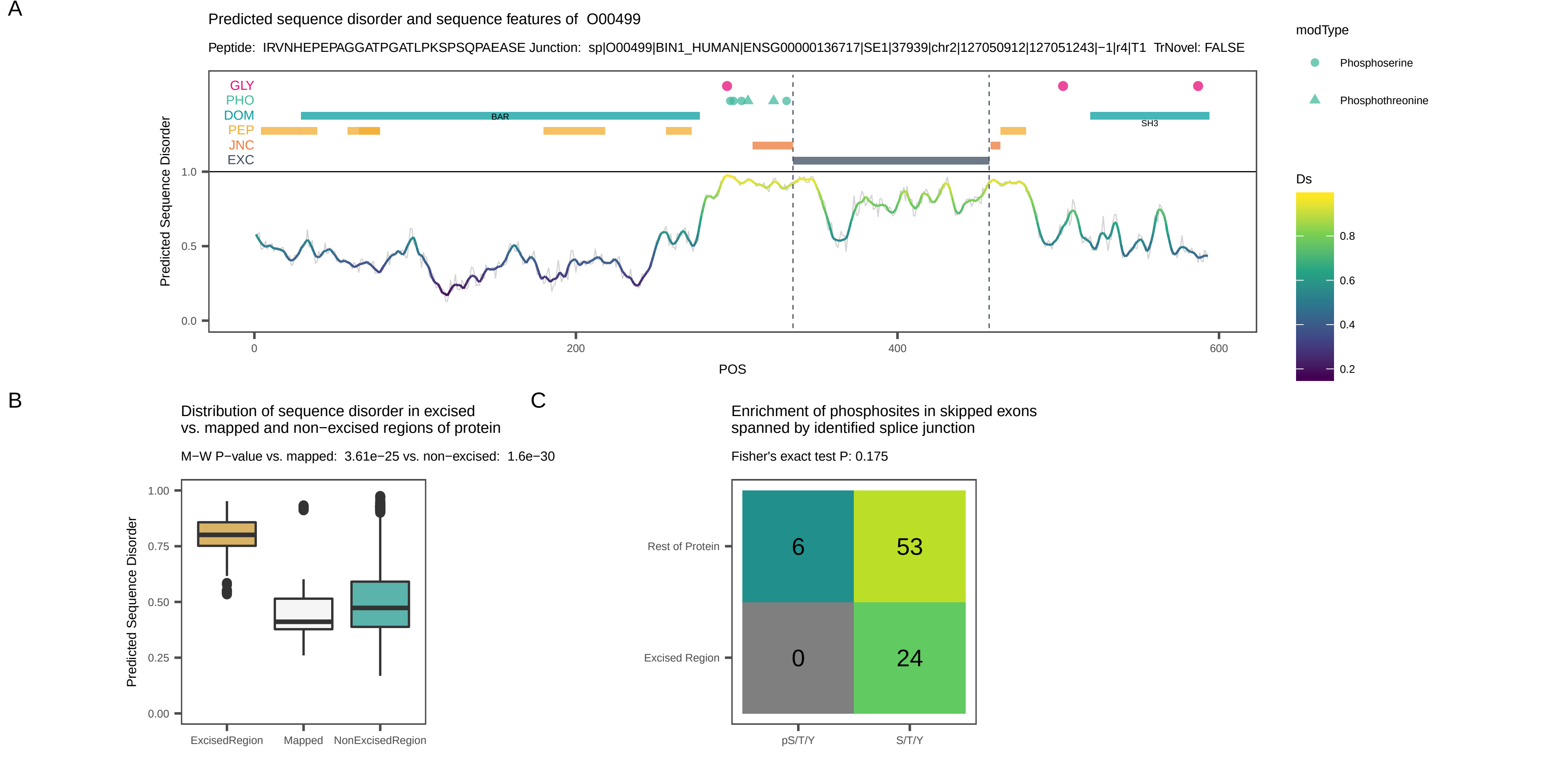

A
Predicted sequence disorder and sequence features of O00499
Peptide: IRVNHEPEPAGGATPGATLPKSPSQPAEASE Junction: sp|O00499|BIN1_HUMAN|ENSG00000136717|SE1|37939|chr2|127050912|127051243|−1|r4|T1 TrNovel: FALSE
GLY
PHO
DOM
PEP
JNC
EXC
BAR
SH3
Predicted Sequence Disorder
1.0
0.5
0.0
0
200
400
600
POS
modType
Phosphoserine
Phosphothreonine
Ds
0.8
0.6
0.4
0.2
B
Distribution of sequence disorder in excised
vs. mapped and non−excised regions of protein
M−W P−value vs. mapped: 3.61e−25 vs. non−excised: 1.6e−30
Predicted Sequence Disorder
1.00
0.75
0.50
0.25
0.00
ExcisedRegion
Mapped
NonExcisedRegion
C
Enrichment of phosphosites in skipped exons
spanned by identified splice junction
Fisher's exact test P: 0.175
Rest of Protein
Excised Region
6
53
0
24
pS/T/Y
S/T/Y
